# The phenotypic diversity in the production performance of Alabio ducks (*Anas platyrhynchos* Borneo) in South Kalimantan, Indonesia

**DOI:** 10.5455/javar.2023.j676

**Published:** 2023-06-30

**Authors:** Abrani Sulaiman, Herliani Herliani, Bambang Langai, Parwanto Parwanto, Rendra Surya, Ahmad Iqbal, Gamaliel Simanungkalit

**Affiliations:** 1 Department of Animal Science, Faculty of Agriculture, Lambung Mangkurat University, Banjarbaru, Indonesia; 2 Centre for Carbon Water and Food, School of Life and Environmental Sciences, The University of Sydney, Brownlow Hill, Australia

**Keywords:** Alabio duck, phenotypic characteristics, principal component analysis, body, eggs

## Abstract

**Objective:**

This study aimed to examine the diversity of phenotypic characteristics of female Alabio ducks (*Anas platyrhynchos* Borneo) and their eggs in South Kalimantan, Indonesia.

**Materials and Methods:**

A total of 200 18-month-old ducks and 300 eggs were selected using a survey method with multistage random sampling. These samples were obtained from two districts in the province of South Kalimantan [Banjar (BJ) districts and Tanah Laut (TL)]. The observed data were analyzed descriptively with variations using the independent *t*-test for each location. Principal component analysis (PCA) was deployed to assess the overall variance and define variables with greater discriminatory power between individuals.

**Results:**

The observations on the body’s physical characteristics and the eggs of Alabio ducks showed significant differences between ducks from BJ and TL areas (*p* < 0.05) except for yolk diameter, albumen height, albumen index, and Haugh unit (HU) (*p *> 0.05). Most egg quality traits from BJ were greater than those from TL, which include egg weight, egg length, egg width, yolk weight, albumen weight, shell weight, shell thickness, and egg shape index. The PCA revealed medium-to-high communalities in the phenotypic body characteristics of Alabio ducks and their eggs.

**Conclusion:**

Significant physical characteristics and egg quality differences were found between the two locations, except for the albumen index and HU, with substantial variability within each trait. These traits could explain the total variation in the phenotypic characteristics of female Alabio ducks.

## Introduction

The Alabio duck (*Anas platyrhynchos* Borneo) is a natural genetic resource that has the potential to be a superior type of laying duck in South Kalimantan. The population of ducks in South Kalimantan in 2021 was 4,291,895 heads spread across 13 districts, with an annual egg and meat production of 35,708 and 1,435 tons, respectively [1]. Therefore, Alabio ducks have enormous potential to be developed as broilers and laying ducks. From the aspect of productivity, the highest percentage of egg production is obtained from the intensive system (91%), followed by the semi-intensive system (83%), and then the extensive system (55%) [2].

Apart from genetically possessing high egg production, Alabio ducks are recognized for producing high-quality eggs based on egg weight, shell color, and bright yolk color [3]. Their ability to produce eggs during a specific period varies greatly, and their genetic diversity is predicted to be greater than what is known. However, selection and breeding efforts for Alabio ducks are currently lacking. A few unpublished studies suspected that Alabio ducks experienced a decline in genetic quality, both in production quantity and quality, due to increased inbreeding caused by mating between close relatives [4]. On the other hand, a crucial issue that is currently happening is the threat to the purity of the Alabio duck by the introduction of various duck breeds from different regions, such as Mojosari ducks, Tegal ducks, and Pekin ducks, and also the occurrence of crossbreeding without strict and directed management. As a result, it is anticipated that the authenticity or purity of the Alabio duck will gradually decline and eventually become extinct.

Observation of national character is an urgent necessity in conserving livestock genetic resources [5], which provides important information on the sustainable management of local livestock breeds and represents genetic diversity between nations [5]. This genetic diversity can increase the productivity and uniformity of existing ducks. Furthermore, genetic diversity is essential for forming a nation or livestock clump. Also, identifying and characterization of the local duck breed is very much needed as primary germplasm data and to support the local duck breeding program [6].

Research on Alabio ducks for breeding is incipient as it is time-consuming and technically prohibitive. Genetic improvement efforts can be made through selection and crossbreeding separately or in combination. Research on the phenotypic character of the body or eggs of ducks, including Alabio ducks, has been studied with limitations by Hariyono et al. [7] and Maharani et al. [6]. One way to determine the determinants of duck characteristics is by measuring body parts (morphometrics) and identifying qualitative and quantitative traits [8]. Muzani et al. [9] stated that genetic research to determine body measurements of birds could be done by measuring the parts of the bone, while Narinc et al. [10] argued that the body size and shape of poultry could be used to determine growth standards. However, studies on the variability among physical and egg characteristics in Alabio ducks are limited.

This study aimed to scrutinize the diversity of phenotypic characteristics of female Alabio ducks and their eggs, including body weight, body length, body height, neck length, chest width, abdomen width, pubic width, shank length, beak length and width, beak color, shank color, egg weight, egg shape index, yolk index, albumen index, yolk weight, albumen weight, shell weight, shell thickness, and Haugh units (HUs). The results of this study are expected to serve as primary data for the conservation, selection, and breeding programs of Alabio ducks so that their genetic purity is maintained. Also, the specific phenotype characters can be used as the basis for selection to develop breeding programs in the future.

## Materials and Methods

### Ethical approval, experimental sites, and animals

Procedures and research protocols were approved by the Faculty of Agriculture, Lambung Mangkurat University (#FP-ULM 010621) under Indonesian Government Regulation No. 95 of 2012 on Veterinary Public Health and Animal Welfare. The research was conducted in Banjar (BJ) Regency (3°18’31.0”S 115°00’30.6”E) and Tanah Laut (TL) Regency (3°46’11.3”S 114°48’35.7”E) of South Kalimantan province. The materials used in this study were 200 female Alabio ducks aged between 16 and 18 months. To observe the physical characteristics of their egg quality, 300 eggs were selected from the 200 female ducks and further analyzed in the Animal Science Laboratory at Lambung Mangkurat University.

### Observed variables 

A survey method with multistage random sampling was used to select the 200 female ducks and 300 eggs across the two locations (BJ and TL). The phenotypic measurements were undertaken by means of a digital scale (Pioneer^TM^ Precision, Ohauss^®^, Port Melbourne, Victoria), a digital caliper (500-196-30, Mitutoyo^® ^American Corporation, Aurora, Illinois), a digital micrometer (293-240-30, Mitutoyo^® ^American Corporation, Aurora, IL), and a Roche yolk color fan (RYCF) (YolkFan^TM^, DSM, Heerlen, The Netherlands). The physical phenotype data were as follows:

1. Body weight (gm).
2. Beak length (cm) was the distance from the maxilla’s base to the maxilla’s tip.
3. Beak width (cm) was measured from the left to the right outer edges of the beak.
4. Neck length (cm) was measured from the first to the last cervical vertebrae.
5. Body length (cm) was measured from the neck’s tip to the tail’s base.
6. Body height (cm) was measured from the bottom of the feet to the top of the back.
7. Chest width (cm) was measured at the widest part of the chest.
8. Abdominal width (cm) was measured from the tip of the sternum to the pubic bone.
9. Pubic width (cm) was measured between the right and left pubic bones.
10. Shank length (cm) was measured along the tarsometatarsus (shank).
11. Shank color was measured by comparing the color with RYCF (1–16).
12. Beak color was measured by comparing the color with the RYCF (1–16).

The egg quality measurements were listed as follows:

1. Egg weight (gm).
2. Egg yolk weight (gm).
3. Dry shell weight (gm).
4. Albumen weight (gm).
5. Egg length (mm).
6. Egg width (mm).
7. Yolk diameter (mm).
8. Albumen diameter (mm).
9. Egg yolk color (1–16).
10. Shell thickness (mm).
11. Egg shape index.
12. Yolk index.
13. Albumen index.
14. HU.

The HU was calculated based on the following formula:

HU = 100 log (*H* + 7.57 − 1.7 *W* ^0.37^)

where *H* is albumen height (mm) and *W* is egg weight (gm) [11].

### Data analysis

Observational data that has been collected is then grouped according to its classification. Data, which is a parameter of physical phenotypic characters for the body of Alabio ducks and their eggs, were analyzed by the independent sampling *t*-test to distinguish two locations (BJ and TL). Principal component analysis (PCA) was determined separately for each location, representing a linear combination of the available variables into a factor or component. Next, Kaiser-Meyer-Olkin (KMO) was used to measure the sampling adequacy of each variable. Following this, Bartlett’s test of sphericity was determined to test the validity of the factor analysis of each data set. Before finding the rotated component matrix, the eigenvalues, percentage of the total variance, and communalities of the body’s quantitative traits were measured. Data analysis was performed using SPSS v.21.0 software (SPSS Inc., Chicago, IL, 2012).

## Results 

### Physical characteristics

The physical phenotypic characteristics of the Alabio ducks for both locations are described in Table 1. The body measurements of Alabio ducks from BJ were relatively uniform, with a coefficient of variation (CV) of <10%, except for body weight (11.91%) and pubic width (28.06%). In contrast, the physical characteristics of Alabio ducks from TL were diverse, with four parameters having a CV > 10%. There was a significant difference in the bodily phenotypic traits of Alabio ducks between BJ and TL across all parameters (*p* < 0.05) (Table 1).

### Egg characteristics

The phenotypic characteristics of Alabio duck eggs across BJ and TL areas are described in Table 2. The traits of Alabio duck eggs for BJ are generally uniform with a relatively small CV (<15%) except for albumen height (26.2%), albumen index (32.0%), and HU (20.3%). The characteristics of Alabio duck eggs for TL are likewise uniform except for albumen height (19.2%) and albumen index (24.7%). However, the traits of Alabio duck eggs between BJ and TL were significantly different (*p* < 0.05) except for yolk diameter, albumen height, albumen index, and HU (*p* > 0.05). The results showed that the egg traits from BJ were greater than those from TL in egg weight, egg length, egg width, yolk weight, albumen weight, shell weight, shell thickness, and egg shape index. On the contrary, TL was superior to BJ in yolk height, yolk color, and yolk index (*p* < 0.05) (Table 2).

**Table 1.** Diversity of physical characteristics of Alabio duck in South Kalimantan from two different locations.

Body characters^*^	Mean ± SD	Location	Mean ± SD	CV (%)
Body weight (gm)	1,418.0 ± 168.58	BJ	1,341.7 ± 159.83^ a^	11.91
		TL	1,494.2 ± 140.83^ b^	9.43
Beak length (cm)	6.2 ± 0.35	BJ	6.4 ± 0.23^ b^	3.57
		TL	6.0 ± 0.26^ b^	4.37
Beak width (cm)	2.7 ± 0.16	BJ	2.6 ± 0.07^ a^	2.73
		TL	2.8 ± 0.14^ b^	5.03
Neck length (cm)	16.4 ± 1.08	BJ	16.3 ± 0.91^ a^	5.60
		TL	16.6 ± 1.22^ b^	7.35
Body length (cm)	20.2 ± 2.61	BJ	17.9 ± 0.82^ a^	4.57
		TL	22.5 ± 1.54^ b^	6.83
Body height (cm)	24.3 ± 1.64	BJ	23.0 ± 0.85^a ^	3.70
		TL	25.5 ± 1.24^ b^	4.86
Chest width (cm)	8.4 ± 0.51	BJ	8.2 ± 0.52^ a ^	6.31
		TL	8.7 ± 0.42^ b^	4.86
Abdomen width (cm)	8.9 ± 1.09	BJ	8.2 ± 0.8^ a^	9.75
		TL	9.5 ± 0.96^ b^	10.11
Pubic width (cm)	3.7 ± 1.41	BJ	2.7 ± 0.77^ b^	28.06
		TL	4.7 ± 1.24 ^a^	26.58
Shank length (cm)	4.6 ± 0.54	BJ	5.0 ± 0.24^ b^	4.83
		TL	4.3 ± 0.53^ a^	12.38
Shank color	14.4 ± 0.72	BJ	14.7 ± 0.57^ b^	3.89
		TL	14.1 ± 0.74^ a^	5.24
Beak color	10.6 ± 3.68	BJ	13.4 ± 1.00^ b^	7.48
		TL	7.8 ± 3.24^ a^	41.65

* Different superscript letters on the same physical character show significant differences (*p* < 0.05).

*n* = 200 female ducks; CV = Coeficient of variation; BJ = Banjar regency; TL = Tanah Laut regency.

### Principal component analysis 

The PCA results are shown in Tables 3 and 4. The KMO measure of sampling adequacy computed for body measurements of Alabio ducks either in BJ or TL was found to be 0.75 and 0.56, respectively. The correlation matrices tested with Bartlett’s test of sphericity for quantitative body traits of the Alabio ducks were significant for BJ (*X*^2^ = 374.67; *p* < 0.001) and TL (*X*^2^ = 262.12; *p* < 0.001). The rotated component matrix, the eigenvalues of the total variance, and communalities for all quantitative traits in the investigated duck populations are also presented in those tables.

**Table 2.** Diversity of egg characteristics of Alabio duck in South Kalimantan from two different locations.

Egg characters^*^	Mean ± SD	Location	Mean ± SD	CV (%)
Egg weight (gm)	66.0 ± 4.98	BJ	69.4 ± 4.57^ b^	6.59
		TL	66.0 ± 4.98^ a^	7.55
Egg length (mm)	57.4 ± 2.19	BJ	61.5 ± 2.99^ b^	4.86
		TL	57.4 ± 2.19^ a^	3.82
Egg width (mm)	44.9 ± 3.46	BJ	49.4 ± 2.40^ b^	4.86
		TL	44.9 ± 3.46^ a^	7.72
Yolk height (mm)	18.8 ± 1.50	BJ	17.1 ± 1.99^ a^	11.62
		TL	18.8 ± 1.50^ b^	7.99
Yolk diameter (mm)	46.1 ± 2.05	BJ	45.8 ± 4.01	8.76
		TL	46.1 ± 2.05	4.45
Albumen height (mm)	6.0 ± 1.16	BJ	6.2 ± 1.62	26.17
		TL	6.0 ± 1.10	19.18
Albumen diameter (mm)	64.8 ± 6.41	BJ	67.2 ± 9.51^ b^	14.15
		TL	64.8 ± 6.41^ a^	9.89
Yolk weight (gm)	23.3 ± 2.39	BJ	24.4 ± 3.12^ b^	12.82
		TL	23.3 ± 2.39^ a^	10.27
Shell weight (gm)	6.2 ± 0.51	BJ	6.5 ± 0.60^ b^	9.21
		TL	6.18 ± 0.51^ a^	8.27
Shell thickness (mm)	0.4 ± 0.03	BJ	0.43 ± 0.04^ b^	9.02
		TL	0.36 ± 0.03^ a^	8.94
Yolk color	15.0 ± 0.16	BJ	14.0 ± 1.14^ a^	8.18
		TL	15.0 ± 0.16^ b^	1.08
Albumen weight (gm)	36.5 ± 3.51	BJ	38.5 ± 3.95^ b^	10.28
		TL	36.5 ± 3.51^ a^	9.61
Egg shape index	0.78 ± 0.06	BJ	0.8 ± 0.03^ b^	4.11
		TL	0.8 ± 0.06^ a^	8.05
Yolk index	0.41 ± 0.03	BJ	0.4 ± 0.06^ a^	14.61
		TL	0.4 ± 0.04^ b^	8.58
Albumen index	0.09 ± 0.02	BJ	0.09 ± 0.03	32.04
		TL	0.09 ± 0.02	24.63
HU	73.4 ± 10.00	BJ	72.6 ± 14.72	20.26
		TL	73.4 ± 10.00	13.62

* Different superscript letters on the same egg character show significant differences (*p* < 0.05).

*n* = 200 female ducks; CV = Coefficient of variation; BJ = Banjar regency; TL = Tanah Laut regency.

For quantitative body traits of the Alabio ducks, the communalities representing estimates of the variance in each variable observed ranged between 0.538 (shank length) and 0.805 (neck length) for BJ, between 0.532 (body height) and 0.855 (pubic width) for TL. In Alabio ducks from BJ, the principal components accounted for 66% (PC1–PC4) of the total variance in the original variables measured, with eigenvalues of 3.90, 1.78, 1.28, and 1.01, respectively. PC1 had high loadings on body weight, chest width, body height, abdomen width, and pubic width, and PC2 had high loadings on body length, beak length, and beak width. In addition, PC3 had high loadings on the beak and shank color, and PC4 had high loadings on the neck and shank length. In Alabio ducks from TL, the principal components accounted for 69% (PC1–PC5) of the total variance in the original variables measured, with eigenvalues of 2.47, 1.88, 1.67, 1.19, and 1.07, respectively. PC1 had high loadings on body length, chest width, body height, and shank length, whereas PC2 had high loadings on body weight, abdomen width, and pubic width. In addition, PC3 had high loadings on the beak and shank color, whereas PC4 had high loadings on the beak length, width, and neck length.

**Table 3.** Rotated component matrix, communalities, eigenvalues, and percentage of the total variance of Alabio ducks’ body measurements in South Kalimantan.

Trait	Location	Principal component					Communalities
		PC1	PC2	PC3	PC4	PC5	
Body weight (gm)	BJ	0.789					0.768
	TL		0.739				0.794
Beak length (cm)	BJ		0.534				0.593
	TL				0.753		0.651
Beak width (cm)	BJ		0.718				0.575
	TL				0.816		0.736
Neck length (cm)	BJ				0.884		0.805
	TL					0.919	0.847
Body length (cm)	BJ		0.738				0.703
	TL	0.598					0.564
Body height (cm)	BJ	0.756					0.636
	TL	0.685					0.532
Chest width (cm)	BJ	0.722					0.598
	TL	0.598					0.537
Abdomen width (cm)	BJ	0.732					0.593
	TL		0.686				0.734
Pubic width (cm)	BJ	0.804					0.688
	TL		0.885				0.855
Shank length (cm)	BJ				0.524		0.538
	TL	0.700					0.605
Shank color	BJ			0.832			0.724
	TL			0.824			0.733
Beak color	BJ			0.821			0.745
	TL			0.823			0.703
Eigenvalues	BJ	3.90	1.78	1.28	1.01		
	TL	2.47	1.88	1.67	1.19	1.07	
% of variance	BJ	32.54	14.81	10.65	8.39		
	TL	20.62	15.64	13.95	9.93	8.94	

BJ = Banjar regency; TL = Tanah Laut regency.

**Table 4.** Rotated component matrix, communalities, eigenvalues, and percentage of the total variance of Alabio ducks’ egg measurements in South Kalimantan.

Trait	Location	Principal component						Communalities
		PC1	PC2	PC3	PC4	PC5	PC6	
Egg weight (gm)	BJ			0.916				0.918
	TL	0.954						0.962
Egg length (mm)	BJ						0.712	0.879
	TL	0.805						0.771
Egg width (mm)	BJ				0.720			0.698
	TL					0.889		0.915
Yolk height (mm)	BJ				0.694			0.715
	TL					0.757		0.899
Yolk diameter (mm)	BJ			0.604				0.577
	TL	0.615						0.721
Albumen height (mm)	BJ		0.963					0.953
	TL		0.956					0.955
Albumen diameter (mm)	BJ			0.532				0.705
	TL		0.495					0.641
Yolk weight (gm)	BJ	0.817						0.944
	TL	0.659						0.945
Shell weight (gm)	BJ					0.809		0.962
	TL				0.846			0.944
Shell thickness (mm)	BJ				0.897			0.824
	TL				0.819			0.768
Yolk color	BJ				0.442			0.390
	TL					0.510		0.504
Albumen weight (gm)	BJ	0.753						0.963
	TL	0.837						0.981
Egg shape index	BJ					0.905		0.901
	TL					0.952		0.941
Yolk index	BJ				0.754			0.825
	TL						0.923	0.939
Albumen index	BJ		0.898					0.926
	TL		0.925					0.976
HU	BJ		0.963					0.948
	TL		0.950					0.945
Eigenvalues	BJ	5.48	3.15	2.46	1.99	1.62	1.30	
	TL	5.07	3.89	2.75	2.00	1.70	1.29	
% of variance	BJ	28.85	16.58	12.93	10.46	8.53	6.85	
	TL	26.67	20.47	14.48	10.53	8.97	6.79	

BJ = Banjar regency; TL = Tanah Laut regency.

For egg quantitative traits of the Alabio ducks, the communalities representing estimates of the variance in each variable observed ranged between 0.390 (yolk color) and 0.980 (percentage of albumen) for BJ, between 0.504 (yolk color) and 0.981 (albumen weight) for TL. The principal components by PCA accounted for 84% and 88% (PC1–PC6) of the total variance for BJ and TL, respectively, with eigenvalues between 1.30 and 5.48 for BJ and between 1.29 and 5.07 for TL. In BJ, PC1 had high loadings on yolk weight, albumen weight, and percentage of yolk and albumen, whereas, in TL, PC1 had high loadings on egg weight and length, yolk diameter, yolk weight, and albumen weight.

## Discussion

Alabio ducks possess different phenotypic characteristics and behaviors than other local ducks in Indonesia. This fact is corroborated by the fact that ducks, particularly in South Kalimantan, have diverse qualitative and quantitative traits [2]. According to Suparyanto [12], phenotypic variations in ducks are partly due to the intensity of external crossbreeding in an unstructured manner, even though one of the broodstock sources is still one family. These variations can be analyzed using the PCA [13].

Suryana [13] found differences in Alabio ducks in three other regencies of South Kalimantan with body weight, beak length, beak width, neck length, and body length of 1.6 gm, 5.5, 2.2, 21.6, and 22.1 cm, respectively. Maharani et al. [6] found that the beak length, beak width, neck length, and shank length were 5.9, 2.8, 17.1, and 6.6 cm, respectively. These differences are attributed to age and environmental factors, including feed. Suryana et al. [14] contended that the difference in phenotypic characteristics was because ducks received different feed ingredients and nutritional values in their rations. This study’s results align with those of Sopiyana et al. [15], who found that the difference in body weight and size between Tegal, Magelang, and Damiaking ducks was influenced by different feeding management.

The beak and shank color are distinctive characteristics of the Alabio duck compared to other Indonesian local ducks. Based on the RYCF score, the average color for the beak is 10.58, and the shank color is 14.4. According to Sulaiman and Rahmatullah [2], 48% of the beak color samples ranged between 6 and 15, and 86% of the shank samples were between 6 and 15. The difference in beak color was mainly due to the age of production. The yellow color of the beak would fade in concert with the increase in the age of the laying period. In this present experiment, the beak color at the first molting (18 months) was <8, while the shank color was relatively similar (>14).

The observed traits contained in the same principal component were classified together in the same group, which may have the same genomic site for their genetic control. The results imply important biological aspects underlying the association between the observed phenotypic traits [6]. Ogah et al. [16] reported that the body size of ducks has a large variance and has a high positive correlation among shank length, beak width, body length, body width, neck length, and head length. Further, Maharani et al. [6] concluded that the three PCA generated from their research could be useful for animal selection and genetic improvement of a particular trait.

Eggs are a poultry farming product with complete nutritional content and are easy to digest. In general, an egg consists of three main components: the shell (11%), albumen (57%), and yolk (32%) [17,18]. In this current experiment, the average egg weight was 69 ± 4.6 (BJ) and 66 ± 5.0 gm (TL). These results align with those of Sulaiman and Rahmatullah [2] (63.80–66.38 gm). Ismoyowati and Purwantini [19] reported that the egg weights of Bali ducks and Alabio Ducks are relatively the same (66.7 *vs.* 65.74 gm) within the range of average duck egg weights (60–70 gm). According to Bell [20], egg weight is divided into four classes, namely jumbo (>63.8 gm), large (56.7–63.7 gm), medium (49.6–56.6 gm), and small (<49.6 gm). Factors that affect egg weight, length, and width are the environment, age of hens, egg composition, and egg-laying period [18].

This current experiment showed that the egg shape index was 0.80 (BJ) and 0.78 (TL). This result was in line with Okatama et al. [21], who found that the normal egg-shaped index of ducks was between 0.70 and 0.79. Likewise, Haryanto et al. [22] reported that the average egg-shaped index was 0.78. Although several factors affect the egg shape index, including origin, production status, genetics, and individual and group variations, the hens’ body weight also affects the egg’s shape. The higher the index, the more round the egg will be, while the lower the index, the more oval the egg [23].

On average, the yolk weight is 23.3 gm (35%), and the albumen weight is 36.5 gm (55%). These results are relatively similar to the previous study by Ismoyowati and Purwantini [19], where the yolk weight was 23.5 gm and the albumen weight was 33.5 gm, with yolk and albumen indexes of 0.41 and 0.0942, respectively. The yolk formation process produces different egg yolk weights depending on the genetic ability of each bird and nutrient consumption. The yolk is formed 10–12 days before the hen lays eggs. The yolk weight ranges from 30% to 33% of the total egg weight [24]. The difference in egg white weight is due to differences in the ability of each duck to synthesize egg white [19]. The amount of egg white synthesis and secretion varies depending on the amount of egg white synthesis in each bird [25].

The bright yellow color of the yolk is the preferred color for consumers. The yolk color obtained in this present study is relatively similar to that obtained by Ismoyowati and Purwantini [19] (15.0 *vs. *14.9) but higher than that reported by Sulaiman and Rahmatullah [2] (10.24–12.54). This is because the color of the yolk is primarily determined by the feed, which is rich in carotene and other pigments [24]. Although consumer perception of egg yolk color is generally linked to geographical location, Hernandez et al. [26] stated that culture and traditions make it true that consumers in most parts of the world prefer deeply hued yolks. Moreover, they pointed out that yolk color in laying hens is primarily determined by the content and profile of pigmenting carotenoids present in their feed and can be easily adapted via feed ingredients.

The HU reported in this current study is 72.6 (BJ) and 73.4 (TL). These values are lower than those obtained by Sulaiman and Rahmatullah [2] (75.1–77.6) and Ismoyowati and Purwantini [19] (78.1). HU is the freshness value of an egg, generally influenced by the length of storage and the egg storage environment. Caner [27] stated that HU is the quality of albumen, which is measured based on the height of the egg white and egg weight. Therefore, the HU value is highly dependent on albumen height, and HUs decrease with storage time, and this decrease occurs more quickly at higher temperatures [18].

There was a significant difference in shell thickness between the two locations (0.43 and 0.36). Sulaiman and Rahmatullah [2] found the shell thickness of Alabio ducks ranged between 0.35 and 0.37 mm, while Ismoyowati and Purwantini [19] reported an average shell thickness of 0.43 mm. Leach Jr. and Gross [28] stated that the eggshell layer calcification is divided into mammillary, palisade, and crystal surface layers. Differences in eggshell thickness in poultry are influenced by genetics, feed, age, and environmental temperature. In addition, adult hens can only store a certain amount of calcium in the eggshell, which is also influenced by genetics and the bird’s age. According to Roberts [18], factors affecting the external quality of eggs (egg size, shell weight, and shell thickness) and the internal quality of eggs (yolk color, albumen quality) are strain, age, nutrition, consumption, disease, molting, stress, storage time, and water quality.

Tuiskula-Haavisto et al. [29] found that the characteristics of egg production influence quantitative trait loci, affecting the age at first laying eggs, egg weight, and the number of eggs on the Z chromosome. Apart from genetic factors, the variability among the physical and egg quality traits of alabio ducks found in this study was likely due to environmental factors, mainly feeds, production management, and temperature conditions. In the BJ district, most Alabio ducks are reared near the river, which is the most suitable habitat for ducks and other waterfowl. On the other hand, the TL district is considered a highland where the altitude above sea level is higher than the BJ district. Huang and Lin [30] contended that environmental factors highly influence the production performance of ducks. Further studies are required to investigate the traits’ variability, repeatability, and heritability under similar environmental conditions.

## Conclusion

Although the physical characteristics of Alabio ducks and their eggs have similar phenotypic characteristics, significant differences were found between the two locations, except for the albumen index and HU, with substantial variability within each trait. The PCA revealed medium to high communalities in the phenotypic characteristics of the body of Alabio ducks and their eggs, indicating that these traits could explain the total variation in the phenotypic characteristics of female Alabio ducks. These results are expected to be a complementary database that can be used as the basis for the Alabio duck breeding program.

## List of abbreviations

BJ: district of Banjar; CV: coefficient of variation; HU: Haugh Unit; KMO: Kaiser-Meyer-Olki; PCA: principal component analysis; RYCF: roche yolk color fan; TL: district of Tanah Laut.
